# Assessment of Bacterial Load and Post-Endodontic Pain after One-Visit Root Canal Treatment Using Two Types of Endodontic Access Openings: A Randomized Controlled Clinical Trial

**DOI:** 10.3390/dj12040088

**Published:** 2024-04-01

**Authors:** Ahmed M. Al-Ani, Ahmed H. Ali, Garrit Koller

**Affiliations:** 1 Aesthetic and Restorative Dentistry Department, College of Dentistry, University of Baghdad, Baghdad 10071, Iraq; ahmed.mothana1104a@codental.uobaghdad.edu.iq; 2 Conservative and MI Dentistry (Including Endodontics), King’s College London Dental Institute at Guy’s Hospital, King’s Health Partners, London SE1 9RT, UK; garrit.koller@kcl.ac.uk; 3 Centre for Oral, Clinical and Translational Sciences, Faculty of Dentistry, Oral and Craniofacial Sciences, King’s College London, London WC2R 2LS, UK; 4 London Centre for Nanotechnology, London WC1H 0AH, UK

**Keywords:** root canal treatment, traditional endodontic cavity, conservative endodontic cavity, colony-forming units

## Abstract

The need for controlling bacteria and pain during root canal therapy is undeniable. This clinical trial aimed to assess whether there is a difference in colony-forming unit (CFU) reduction after instrumentation and post-endodontic pain after root canal treatment (RCT) using a traditional endodontic cavity (TEC) versus a conservative endodontic cavity (CEC). This clinical study was conducted on 89 patients designated for a single-visit RCT. Patients were allocated randomly (TEC *n* = 45 and CEC *n* = 44). The access opening was gained accordingly in each group by a single operator. A pre-instrumentation sample of root canal dentin was collected using an endodontic file; the second sample was collected similarly, right after shaping and cleaning the root canal. The CFU was calculated based on the samples collected. The pain level was recorded preoperatively and at 1, 7, and 21 days postoperatively utilizing a visual analog scale (VAS). There were no statistically significant differences in the CFU reduction between the TEC and CEC groups (*p* > 0.05). Additionally, there were no statistically significant differences found in postoperative pain levels between the TEC and CEC at 1, 7, and 21 days (*p* > 0.05). Despite the limitations of this study, both the CEC and TEC demonstrate a decrease in bacteria within the root canals and alleviate postoperative pain with no difference between them.

## 1. Introduction

The primary objectives of root canal treatment revolve around addressing and preventing apical periodontitis, achieved through thorough cleaning, and the shaping of root canals to eradicate microbes [1,2,3]. Creating access to the pulp chamber and root canal system stands as an initial and crucial stage in effective endodontic treatment [4,5]. A well-prepared access cavity allows for essential subsequent procedures of canal localization, measurement, cleaning, shaping, and sealing [6,7,8].

The traditional endodontic cavity has seen minimal changes over time. This type of access cavity requires removing the chamber roof, reducing dentin protrusions, and widening the canal entrance, to minimize the risk of procedural errors [6]. However, several authors argue that this type of cavity preparation removes a substantial amount of dentin, compromising the tooth’s structure, which requires additional restorative materials, such as posts and cores to restore function and form, which can further weaken the tooth. In addition, this overpreparation increases the need for crowns in the future and this means further structural compromise and additional expenses to the patient [6,9,10]. 

A newer access cavity, a conservative endodontic cavity, prioritizes preserving the dental structure and specifically focuses on maintaining the pulp chamber roof and pericervical dentin situated approximately 4 mm above and below the alveolar crest. Although the apex of the root can be amputated, and the coronal third of the clinical crown removed and replaced prosthetically, this dentin near the alveolar crest is irreplaceable and it is important for the ferrule and fracture strength, as the long-term retention of the tooth and resistance to fracturing are directly related to the amount of residual tooth structure [9,11].

Microorganisms play a pivotal role in periapical infections related to root canals. Mechanical cleaning of contaminated roots is coupled with various disinfection agents and irrigation protocols to decontaminate infected root canals [12]. Clinicians and researchers aim realistically to reduce bacterial contamination as much as possible, below a level crucial in either inducing or sustaining disease, and this goal is widely accepted [13].

The flora within the root canal consists of various microorganisms, mainly anaerobic ones. These microbes, particularly anaerobes, are frequently linked to issues affecting the pulp and tissues surrounding the roots, and they are often associated with failures in endodontic treatment. These anaerobic organisms have the capability to break down amino acids and polypeptides available to them for metabolic requirements. Among these microbes, *E. faecalis* is frequently identified in persistent endodontic infections without symptoms, due to its possession of numerous traits aiding in survival and virulence, making it a significant contributor to endodontic treatment failures [14,15].

Bacterial endotoxins (lipopolysaccharides (LPS) present in the cell wall of Gram-negative bacteria) elicit different inflammatory reactions in the pulp. Teeth exhibiting symptoms of irreversible pulpitis tend to exhibit elevated levels of these endotoxins. Through binding to cell surface receptors, these endotoxins prompt inflammation within the dental pulp, leading to the production and release of proinflammatory cytokines, nitric oxide, and eicosanoids [16]. The root canal environment (moist, warm, nutrient-rich, and anaerobic) provides microorganisms with ideal conditions for colonization and proliferation, shielding them from host defenses. Detecting bacteria within the root canal space is crucial for determining the necessity of continued disinfection during RCT, aimed at reducing bacterial load to enhance treatment success rates. Various methods are employed for bacterial detection and quantification, including colony-forming unit (CFU) counting and DNA extraction followed by polymerase chain reaction (PCR) and its derivatives, as well as DNA–DNA hybridization techniques [17,18].

The term “post-endodontic pain” describes any uncomfortable feeling or tenderness that transpires after an endodontic treatment. Post-endodontic pain is frequently experienced sensation reported by 3–58% of patients that may persist from a few hours to many days after endodontic therapy [19]. Notably, there is strong evidence indicating a relationship between preoperative and postoperative pain [20]. When preoperative pain is present, there is heightened activation of nociceptive impulses, which significantly raises the likelihood of experiencing postoperative pain. This increased pain experience primarily stems from the inflammatory effects inherent in root canal infections, triggering the activation of nociceptors and subsequent central sensitization. Furthermore, postoperative endodontic pain arises from various factors, including acute inflammation in the periapical region, potentially linked to localized damage from chemical, mechanical, host, or microbial sources during endodontic procedures [21,22].

When debris, organic tissue, microbes, and irrigant solutions are pushed beyond the apical foramen during RCT, they can trigger inflammation and lead to postoperative problems, including mild to severe pain; the intensity of pain may vary depending on the degree of periapical tissue injury. However, the effect of the access-opening type on the apically extruded debris or the outward flushing of the irrigation solution, which in turn affects the pain, could be a matter of concern [23,24,25].

Studying the impact of access-opening types on post-endodontic pain and bacterial reduction is crucial for optimizing endodontic treatment outcomes. Understanding how these access techniques influence patient discomfort, microbial control, and structural integrity can inform clinicians in selecting the most appropriate approach. This comprehensive evaluation addresses various aspects of endodontic care, contributing to enhanced clinical decision making and patient-centered treatment strategies.

This two-arm randomized controlled study aimed to investigate the postoperative pain intensity and to measure bacterial count before and after instrumentation in two groups of patients requiring root canal treatment of posterior teeth accessed by two types of access cavities: TEC and CEC. The null hypothesis stated that there were no differences in postoperative pain intensity in the two groups of patients after a single-visit RCT. Also, there was no difference in bacterial count reduction post-instrumentation in the teeth of the two groups of patients.

## 2. Materials and Methods

### 2.1. Ethics Approval and Consent to Participate

The CONSORT (Consolidated Standards of Reporting Trials) guidelines have been followed for this manuscript. The single-blind (patients were blinded to the type of endodontic access cavity) parallel-arm randomized controlled clinical trial was performed at an endodontic postgraduate dental clinic (College of Dentistry, University of Baghdad) for the period 28 March 2023 to 3 July 2023. The study protocol was approved by the University Ethics Committee (No. 799523) and registered under the ClinicalTrials.gov domain (ID: NCT06027931). Patients were recruited at the endodontic postgraduate dental clinic. Patient information sheets were distributed and written informed consents were obtained from the patients before their enrolment into the study. The study was carried out in compliance with the principles of the Declaration of Helsinki 2013.

### 2.2. Sample Size Calculation

This study was designed to have 80% power with an alpha margin of error of 5%. The sample size was calculated using the G-power software calculator (version 3.1.9.7) to detect an expected difference of 25% in the bacterial count between the two groups; thus, a total sample size of 84 (allocation ration of 1:1) root canal treatments was calculated anticipating a 10% loss at follow-up.

### 2.3. Randomization

A clinician who had not contributed to the study made the randomization centrally using a computer random table generator (http://www.random.org) accessed on 26 March 2023, and each patient received a consecutive number. The tooth was the unit of randomization. The samples were stratified according to the sex and tooth type (molar and premolar). The allocation ratio was 1:1, and the allocation concealment was performed using an envelope-based system.

### 2.4. Inclusion and Exclusion Criteria

The inclusion criteria of patients included mentally, and physically, healthy adults aged 18 years or older, who had not taken analgesics or antibiotics in the week preceding the treatment. Dentally, eligible teeth were permanent mature molars or premolars diagnosed with necrotic pulp or irreversible pulpitis, with or without signs of apical periodontitis, determined through a combination of patient history; comprehensive clinical examination; pulp vitality testing (cold and electrical pulp tester), along with palpation; percussion tests; and radiographic assessment (using periapical radiographs and cone beam computed tomography (CBCT)). The exclusion criteria comprised pregnancy, individuals younger than 18 or older than 65 years, teeth exhibiting internal or external resorption, an open apex, or previous root canal treatment, as well as patients who had used antibiotics or analgesics within the past 7 days.

**Figure 1:**
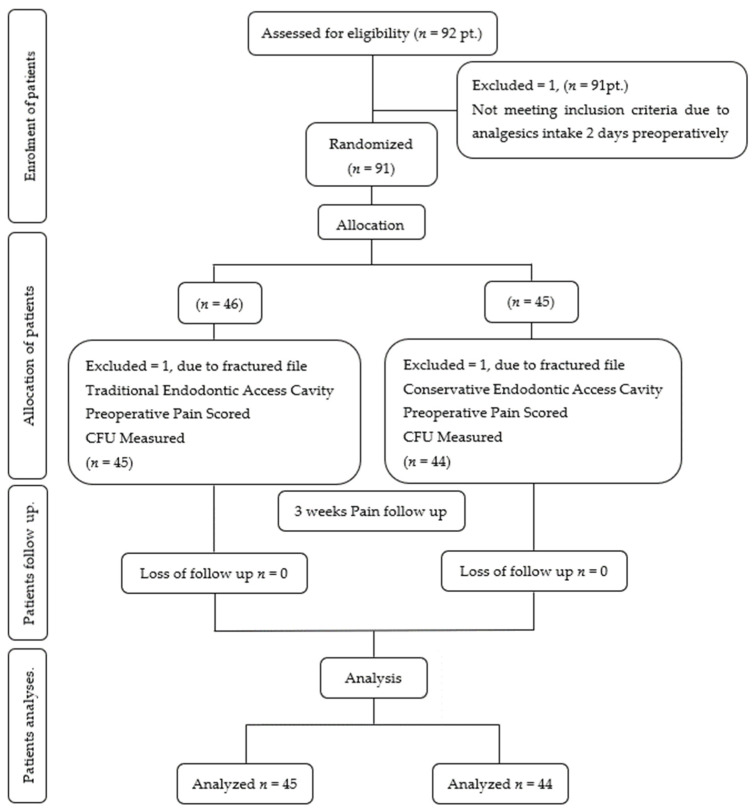
Flow diagram showing patient recruitment and follow-up. Adapted from the CONSORT flow diagram.

A single trained operator, familiarized with extracted teeth, conducted root canal treatments (RCTs) on maxillary/mandibular molars and premolars. Digital periapical radiographs (CS 2100, Kodak, Carestream, Atlanta, GA, USA) with a periapical digital sensor (CS 6500, Kodak, Carestream, Atlanta, United States) and CBCT scans (CS 8100, Kodak, Carestream, Atlanta, United States) with 8 cm × 9 cm FOV, 90 Kv, 10 mA, 14 s exposure time, 5 μSv, and 75 μm voxel size parameters were made for each patient before the RCT. Unless clinically indicated, all RCTs were completed in a single visit. The flowchart outlining the clinical trial’s process is presented in Figure 1.

### 2.5. Clinical Procedure

**Figure 2:**
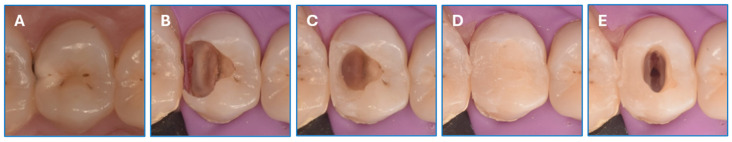
Clinical images of the maxillary right second premolar: (**A**) preoperative; (**B**) after carious tissue removal; (**C**) pre-access cavity re-walling; (**D**) pre-access cavity build-up; (**E**) traditional endodontic access cavity.

**Figure 3:**
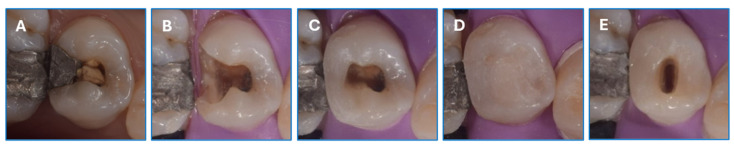
Clinical images of the maxillary right second premolar: (**A**) preoperative; (**B**) after carious tissue removal; (**C**) pre-access cavity re-walling; (**D**) pre-access cavity build-up; (**E**) conservative endodontic access cavity.

After administration of local anesthesia, a rubber dam (Nic Tone, MDC Dental, Belgium) was placed and the singly isolated tooth was cleaned with sterile gauze embedded with 2.5% NaOCl. After removal of caries or previous restoration, without penetration into the pulp cavity, the pre-endodontic build-up was applied with the so-called “Doughnut” technique (Figure 2B and Figure 3B), which involves a circumferential build-up of the cavity walls, and the cavity underwent restoration using resin composite material without performing any access to the pulp chamber. Subsequently, the tooth was re-drilled to establish an endodontic access cavity (TEC or CEC) to access the pulp chamber. Upon completing the root canal treatment in a single session, the space created by the endodontic access cavity was once again filled with resin composite restoration [26]. 

Preparation of the access cavity was accomplished according to each group. In the TEC group, the procedure involved creating an access cavity to the pulp chamber by drilling occlusally using a round diamond bur (Komet, Lemgo, Germany, LOT: 00213371). This was followed by further refining the access cavity and completing the de-roofing of the pulp chamber using an endo Z bur (Dentsply Sirona Endodontics, Ballaigues, Switzerland), in accordance with traditional standards described in established literature. Upon completion of the TEC preparation, the canal orifices became fully visible (Figure 2) [27,28].

In the CEC group, premolars were accessed through the central fossa with dimensions matching the diameter of the round diamond bur (1.2 mm in diameter) (Komet, Lemgo, Germany, LOT: 00213371), with the cavities extended downward while retaining a portion of the chamber roof and lingual shelf. Molars were accessed at the mesial quarter of the central fossa, and the cavities extended both downward and distally, also maintaining a portion of the chamber roof. Minimization of dentin removal in the mesiodistal, buccolingual, and circumferential pericervical areas was prioritized to ensure the preservation of the chamber roof section necessary for clear visualization of all root canal orifices from a single visual perspective [4,28], as shown in Figure 3.

Canal orifices were located, and canal patency was checked with #10 K-file (M. Access, Dentsply, Charlotte, NC, USA). The WL was established with a #15 K-file and the IPEX II electronic apex locator (NSK) and confirmed with a digital radiograph (CS 2100, Kodak, Carestream, Atlanta, United States).

Canals were shaped using crown down techniques with rotary instrumentation using ProTaper Gold rotary files (Dentsply Mallifer, Ballaigues, Switzerland) and the X-Smart IQ Endo Motor (Dentsply) according to a preprogrammed torque and speed. During the cleaning and shaping procedure (which was about 30–35 min for molars and 15–20 min for premolars with some variations according to each case), 5.25% NaOCl was applied using passive irrigation using a 24-gauge needle (Max-I-Probe; Dentsply Sirona) and activated ultrasonically by the Ultra X Ultrasonic Activator (Eighteeth, Changzhou, China) with an ultrasonic tip (#20, taper 2%, 45 kHz) at the end of the instrumentation.

A size #10 K-file was used to maintain WL after each rotary instrument. The apical gauging was performed utilizing a Ni-Ti hand K-file with the same size as the last apical rotating instrument used. The root canals were dried with paper points and filled using the gutta-percha single cone technique (Dentsply Maillefer, Tulsa, OK, USA) and a One-Fil (Mediclus CO., Ltd. Cheongju-si, Republic of Korea) bioceramic root canal sealer.

**Figure 4:**
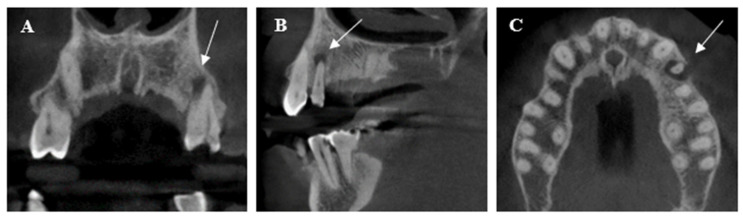
CBCT images showing periapical radiolucency of maxillary left second premolar (white arrow): (**A**) coronal, (**B**) sagittal, and (**C**) axial views.

In our study, we utilized CBCT imaging to capture detailed anatomical structures prior to treatment initiation to assess potential diagnostic concerns such as periapical lesions, missed canals, and other anatomical irregularities. As illustrated in Figure 4, the CBCT image obtained pre-treatment provides a comprehensive view of the patient’s dental morphology.

### 2.6. Bacterial Sampling

Bacterial samples were obtained from the root canals using a strict asepsis procedure. If the root canal was dry, a small amount of sterile saline solution was introduced into the canal. Samples were initially collected using a new, ISO #15 K-file (Dentsply/Maillefer) introduced to a level approximately 1 mm short of the tooth apex, and a discrete filing motion was applied. For the maxillary molars, samples were collected from the palatal root canal, and, for the mandibular molars, samples were collected from the distal root canal. The file was kept in the canal for 1 min, then transferred to test tubes containing 1 mL of phosphate buffer solution. The file’s handle was cut off before insertion into the microtube with a sterilized orthodontic plier to prevent the introduction of external contaminants. Handling of the files was performed with sterile tweezers. The second sample was taken in the same way, right after finishing shaping and cleaning the root canal before the root canal filling [29].

### 2.7. Microbiological Culturing and Analysis

A readily utilized medium, brain heart infusion agar (Oxoid-UK), was prepared according to the manufacturer’s instructions, and then sterilized at 121 °C for 15 min. Tenfold serial dilutions were carried out in saline by mixing 1 mL of a sample with 9 mL parts of saline so that the new solution was 10 times less concentrated than the original solution. This process was repeated to achieve 10 tubes of saline solution containing the diluted sample. Then, aliquots of 1000 μL of each dilution were plated onto BHI agar plates and incubated at 37 °C for 24 h. The colony-forming units that grew were counted and then transformed into actual counts based on the known dilution factors [30].

### 2.8. Pain Level Evaluation

Preoperative pain was recorded on the VAS scale. The VAS scale is a continuous measure comprising a horizontal line, which is 10 cm in length numbered from 0 to 10, each number represents the pain score. For postoperative pain, the patients were asked to put a mark perpendicular to the VAS pain line at the point that indicated their pain intensity during the 1, 7, and 21 days after the endodontic treatment, as performed by Pawar et al. [31].

### 2.9. Statistical Analysis

Data were analyzed using SPSS version 26. Data normality was assessed using the Shapiro–Wilk test. The Mann–Whitney U test was used to compare nonparametric data between groups. Also, the Friedman test and Wilcoxon signed rank test were used to compare within-group data. The significance level was set at *p* < 0.05.

A total of 89 patients were enrolled in this study, with 89 teeth treated. The teeth were randomly allocated to one of the two endodontic access-opening groups (45 patients in the TEC group, and 44 patients in the CEC group). A total of 46 patients (52.3%) were female, and 42 (47.7%) were male. Their ages ranged between 18 and 65 years (age mean was 34); 48 (54.5%) of the treated teeth were molar teeth (25 molars for the TEC group and 24 molars for the CEC group), and 40 (45.5%) were premolar (20 premolars for each group) (Table 1).

## 3. Results

### 3.1. Comparison of Bacterial Count between Techniques, Tooth Type, and Sex

**Figure 5:**
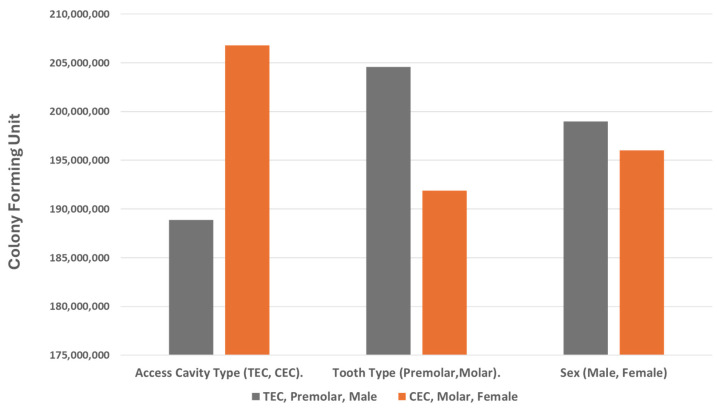
Mean difference in colony-forming units (CFUs) between treatment groups in the clinical trial.

Table 2 summarizes the mean values, standard deviations (SDs), and *p* values associated with the bacterial counts for both the TEC and CEC techniques before and after instrumentation and the difference in counts (see Figure 5). There were no statistically significant differences in the bacterial counts between the traditional and conservative access cavity groups both before and after instrumentation (*p* > 0.05). Additionally, there were no significant differences observed in the bacterial count before and after instrumentation between premolars and molars or between males and females (*p* > 0.05).

### 3.2. Comparison of Pain Level between Techniques, Tooth Type, and Sex

**Figure 6:**
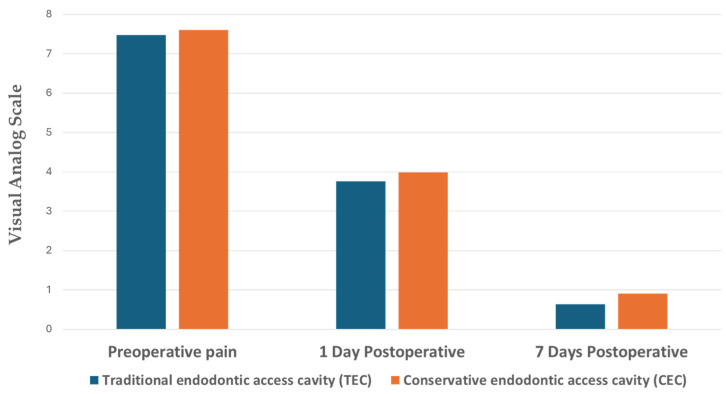
Comparison of the pain level between treatment groups in the clinical trial preoperatively and postoperatively.

Table 3 displays the mean values, standard deviations (SDs), and *p* values of pain levels (see Figure 6). For the TEC group, the mean values of the preoperative pain level and postoperative pain levels at 1, 7, and 21 days were 7.47, 3.76, 0.64, and 0.00, respectively, while for the CEC group, the mean values of the preoperative pain level and postoperative pain levels at 1, 7, and 21 days were 7.61, 3.98, 0.91, and 0.00, respectively. Notably, there were no statistically significant differences found in the preoperative pain level or postoperative pain levels at 1, 7, and 21 days between the TEC and CEC groups (*p* > 0.05). Furthermore, no statistically significant differences were observed based on tooth type (molar or premolar) or gender (male or female) in the preoperative pain level and postoperative pain levels at 1, 7, and 21 days (*p* > 0.05).

### 3.3. Comparison of Bacterial Counts and Pain Level within Groups

There was a significant reduction in the bacterial count between the pre- and post-instrumentation samples in both groups (*p* < 0.001). Also, there was a significant reduction in the pain level from preoperative to postoperative at 1, 7, and 21 days in both groups (*p* < 0.001).

## 4. Discussion

The null hypothesis for this study was accepted, as there were no differences in the postoperative pain levels in the two groups of patients after a single-visit RCT. Also, there was no difference in bacterial count reduction post-instrumentation in the teeth of the two groups of patients.

In this study, all endodontic procedures were conducted by a calibrated experienced single endodontist experienced in rotary techniques as the proficiency of the operator significantly impacts root canal preparation [32]. Molar and premolar teeth were specifically chosen for this study as posterior teeth commonly exhibit the most challenging root canal anatomy and are frequently associated with clinical complications and fractures [33]. In addition, the gutta-percha (GP) bioceramic root canal sealer used, in combination with conventional root canal sealers (e.g., zinc oxide and eugenol cements), might not be able to adequately seal the root canal system; microleakage seems to be inevitable in such clinical situations [34].

Although the conservative approach prioritizes preserving the natural tooth structure whenever possible, it might jeopardize the ability of the clinician to allocate canals because of the restricted accessibility, which mandates using magnification tools. In this study, the operator used Neitz loupes with X5 magnification (NEITZ Instruments, Tokyo, Japan) and there was no incidence of missing the canal in any tooth in either group, which was confirmed with CBCT scan analysis preoperatively. Also, previous studies found no difference between TEC and CEC in the incidence of missing canals [35]. In this study, CBCT was employed due to its superior diagnostic accuracy in detecting missed canals in comparison to periapical radiographs, as it has been reported before that over 85% of root-filled upper first molars have missed canals on CBCT scans [36]. 

There were variations in the cavity size between the molars and premolars and the extent of the tooth decay. Despite these variations, we aimed to ensure an equal representation of premolars and molars between the two groups. Additionally, we applied pre-endodontic build-up before access to the pulp chamber to standardize and minimize the impact of differing destruction levels. This clinical trial aimed to replicate real-life scenarios regarding tooth types and levels of decay, acknowledging the variability encountered in clinical practice.

This study’s findings have consistently suggested that while the access approach may differ the outcomes in terms of bacterial count within the root canals do not notably differ between TEC and CEC access openings before and after instrumentation, indicating that both access types are equally effective in eliminating bacteria from the root canal system, which reflects their enhancement of cleaning and the efficiency and uniformity of instrumentation regardless of the access cavity approach used. When comparing the CEC and TEC, studies have generally reported comparable results concerning untouched canal walls, pulp tissue remnants inside the root canals, or bacteria load reduction in the root canals, which in turn reflects the cleaning ability [37,38,39,40]. However, other studies have reported more untouched canal walls with the CEC versus the TEC [41,42]. 

Despite the absence of statistical differences between the CEC and TEC in bacterial count reduction before and after instrumentation, the skill and experience of the endodontist are crucial factors that may influence the outcomes. A highly proficient endodontist possesses advanced technical abilities, precise handling of instruments, and a thorough understanding of root canal anatomy, enabling them to effectively manage cleaning irrespective of the access cavity type. Studies have indicated that operator expertise significantly impacts the success rates and clinical outcomes of endodontic procedures, including bacterial reduction strategies [43,44,45].

Another factor is that the use of magnification loupes may still have impacted the outcomes. Magnification loupes offer enhanced visualization and precision during endodontic procedures, potentially contributing to more thorough cleaning and shaping of the root canal system and more importantly canal detection and localization. Though not directly influencing the access cavity type comparison, their use could have facilitated more effective bacterial reduction. Studies have highlighted the benefits of magnification in endodontics, emphasizing its role in improving procedural accuracy and outcomes [46].

Regarding the complexity of endodontic infections, it is well recognized that most root canal infections are anaerobically polymicrobial, with a significant presence of obligate anaerobes. In this research, types of bacteria, whether aerobic or anaerobic, may not have significantly impacted the overall reduction achieved. The primary focus of the study was on assessing the general reduction in bacterial count, irrespective of specific bacterial types. Although, to date, there is no surrogate endpoint in clinical settings, bacterial reduction during root canal treatment plays a pivotal role in the success of the treatment. This highlights the importance of considering overall bacterial load reduction as a key outcome measure in evaluating the efficacy of different access cavity techniques. By prioritizing overall reduction rather than specific bacterial types or species, the study provides valuable insights into the effectiveness of access cavity designs in achieving optimal bacterial control during root canal treatment.

Postoperative pain is one of the primary problems in endodontic treatment. The search for existing literature on studies comparing the effects of access cavity design on pain levels yielded no relevant results. Consequently, this study aimed to fill this gap by evaluating how these two distinct access cavity designs, TEC and CEC, impact postoperative pain levels. In this study, postoperative pain was evaluated using a visual analog scale, a method widely accepted for its validity and reliability in endodontic research [47]. To reduce the Hawthorne effect, the potential alteration of behavior due to the awareness of being observed, participants were informed of the study’s objective after they self-recorded their pain levels [48]. This approach aimed to minimize any potential influence on participants’ responses due to their awareness of being part of the investigation.

The study accounted for the impact of premedication on reducing postoperative endodontic pain by excluding participants who had taken medication [49]. Additionally, to minimize the influence of various factors, particularly the effects of intracanal medications and coronal leakage on postoperative pain, a single-visit endodontic treatment approach was employed [50]. This strategy aimed to isolate and focus on specific variables without the confounding influence of multiple treatment sessions or additional factors. Tooth type was also not associated significantly with postoperative pain in this study (*p*-value 0.706). Similar results are found in other studies [51,52]. The extrusion of apical debris might influence the occurrence of postoperative pain [53]. Interestingly, this factor does not appear to rely on the volume of the access cavity (CEC or TEC) [54].

The clinical relevance of this study is that prioritizing the access-opening design should not compromise root canal detection, as missed canals can significantly impact treatment outcomes negatively [55]. Therefore, designing the access opening should follow a thorough preoperative assessment of root canal complexity, possibly through utilizing CBCT.

The limitations of the study included the enumeration of CFU counts from dental hard tissue by traditional methods, like scraping bacteria with endodontic files, primarily capturing accessible bacteria, potentially overlooking those within dense biofilm structures or deep inside dentin tubules. Additionally, previous findings showed dead bacteria penetrating up to around 250 micrometers within dentinal tubules and the endotoxins from dead or devitalized bacteria can trigger inflammation through macrophage infiltration [56]. However, this study focused on assessing the reduction in viable bacteria after cleaning and shaping to understand the impact of cavity type on bacterial counts.

Another limitation was the subjectivity of pain. Being inherently subjective, pain can vary widely among patients due to factors like pain tolerance, psychological state, and previous dental encounters. Despite standardized assessment tools and clinical guidelines, interpretations of pain intensity often rely on patients’ self-reports, which can be influenced by subjective biases. In this study, a visual analog scale was used to assess postoperative pain. This is a valid and reliable method that has been widely used in endodontic literature [57].

## 5. Conclusions

In conclusion, while the traditional and conservative access openings represent different approaches to root canal therapy, this clinical trial demonstrates that there is no notable difference in post-treatment pain levels or bacterial count between the two groups of patients. This challenges the assumption that a larger access opening ensures better outcomes, emphasizing the significance of the practitioner’s skill and technique in achieving successful root canal therapy. The choice between these approaches should be guided by the individual case and the preference for preserving the natural tooth structure while ensuring effective treatment.

In addition, both access cavity techniques appear to be equally effective and viable options in endodontic treatment, and the choice between them may be based on clinician preference and individual patient factors rather than expected differences in treatment outcomes. Further research with larger sample sizes and longer follow-up periods may be warranted to validate these findings and explore additional factors that may influence treatment outcomes.

## Figures and Tables

**Table 1:** Patients’ Demographic Data.

Variables		TEC	CEC	Total
Tooth type	Molar (55%)	25 (51%)	24 (49%)	49
	Premolar (45%)	20 (50%)	20 (50%)	40
Sex	Male	20	23	43
	Female	25	21	46
Age	Age Mean (SD)	32.5 (12.74)	36.4 (13.56)	34.3 (13.24)
Periapical radiolucency	Present	16	17	33
	Absent	28	28	56

**Table 2:** Mean (±SD) of bacterial count difference before and after instrumentation according to the type of access cavity, tooth type, and sex.

Stage	Type of Access Cavity	Mean (SD)	*p*-Value *	Tooth	Mean (SD)	*p*-Value *	Sex	Mean (±SD)	*p*-Value *
Before	TEC	1.892 × 10^8^ (7.846 × 10^7^)	0.356	Premolar	2.050 × 10^8^ (7.671 × 10^7^)	0.331	Male	2.00 × 10^8^ (±6.44 × 10^7^)	0.889
	CEC	2.072 × 10^8^ (6.591 × 10^7^)		Molar	1.923 × 10^8^ (6.932 × 10^7^)		Female	1.97 × 10^8^ (±8.03 × 10^7^)	
After	TEC	3.019 × 10^5^ (5.293 × 10^5^)	0.631	Premolar	3.049 × 10^5^ (5.602 × 10^5^)	0.815	Male	3.92 × 10^5^ (±7.09 × 10^5^)	0.922
	CEC	3.636 × 10^5^ (6.775 × 10^5^)		Molar	3.558 × 10^5^ (6.448 × 10^5^)		Female	2.77 × 10^5^ (±4.89 × 10^5^)	
Difference	TEC	1.889 × 10^8^ (7.846 × 10^7^)	0.362	Premolar	2.046 × 10^8^ (7.674 × 10^7^)	0.336	Male	1.99 × 10^8^ (±6.44 × 10^7^)	0.889
	CEC	2.068 × 10^8^ (6.588 × 10^7^)		Molar	1.919 × 10^8^ (6.926 × 10^7^)		Female	1.96 × 10^8^ (±8.03 × 10^7^)	

* Mann Whitney U test.

**Table 3:** Mean (±SD) of VAS pain level before RCT and after RCT in 1-, 7-, and 21-day periods according to the type of access cavity, tooth type, and sex.

Pain Level Time	Type of Access Cavity	Mean (SD)	*p*-Value *	Tooth	Mean (SD)	*p*-Value *	Sex	Mean (±SD)	*p*-Value *
Preoperative pain	TEC	7.47 (2.92)	0.913	Premolar	7.00 (3.28)	0.114	Male	7.49 (2.61)	0.451
	CEC	7.61 (2.88)		Molar	8.00 (2.44)		Female	7.59 (3.15)	
1 Day	TEC	3.76 (2.66)	0.859	Premolar	3.95 (2.65)	0.706	Male	3.67 (2.99)	0.358
	CEC	3.98 (2.85)		Molar	3.79 (2.85)		Female	4.04 (2.51)	
7 Days	TEC	0.64 (0.96)	0.535	Premolar	0.51 (0.87)	0.082	Male	0.88 (1.35)	0.703
	CEC	0.91 (1.41)		Molar	1.00 (1.40)		Female	0.67 (1.06)	
21 Days	TEC	0.00 (0.00)	1.000	Premolar	0.00 (0.00)	1.000	Male	0.00 (0.00)	1.000
	CEC	0.00 (0.00)		Molar	0.00 (0.00)		Female	0.00 (0.00)	

* Mann Whitney U test.

## Data Availability

Data of the study is available upon request from the authors.
